# Free induction decay navigator motion metrics for prediction of diagnostic image quality in pediatric MRI

**DOI:** 10.1002/mrm.28649

**Published:** 2021-01-06

**Authors:** Tess E. Wallace, Onur Afacan, Camilo Jaimes, Joanne Rispoli, Kristina Pelkola, Monet Dugan, Tobias Kober, Simon K. Warfield

**Affiliations:** ^1^ Computational Radiology Laboratory Department of Radiology Boston Children’s Hospital Boston MA USA; ^2^ Department of Radiology Harvard Medical School Boston MA USA; ^3^ Advanced Clinical Imaging Technology Siemens Healthcare AG Lausanne Switzerland; ^4^ Department of Radiology Lausanne University Hospital and University of Lausanne Lausanne Switzerland; ^5^ LTS5 École Polytechnique Fédérale de Lausanne Lausanne Switzerland

**Keywords:** artifacts, free induction decay navigators, motion detection, pediatric neuroimaging

## Abstract

**Purpose:**

To investigate the ability of free induction decay navigator (FIDnav)‐based motion monitoring to predict diagnostic utility and reduce the time and cost associated with acquiring diagnostically useful images in a pediatric patient cohort.

**Methods:**

A study was carried out in 102 pediatric patients (aged 0‐18 years) at 3T using a 32‐channel head coil array. Subjects were scanned with an FID‐navigated MPRAGE sequence and images were graded by two radiologists using a five‐point scale to evaluate the impact of motion artifacts on diagnostic image quality. The correlation between image quality and four integrated FIDnav motion metrics was investigated, as well as the sensitivity and specificity of each FIDnav‐based metric to detect different levels of motion corruption in the images. Potential time and cost savings were also assessed by retrospectively applying an optimal detection threshold to FIDnav motion scores.

**Results:**

A total of 12% of images were rated as non‐diagnostic, while a further 12% had compromised diagnostic value due to motion artifacts. FID‐navigated metrics exhibited a moderately strong correlation with image grade (Spearman's rho ≥ 0.56). Integrating the cross‐correlation between FIDnav signal vectors achieved the highest sensitivity and specificity for detecting non‐diagnostic images, yielding total time savings of 7% across all scans. This corresponded to a financial benefit of $2080 in this study.

**Conclusions:**

Our results indicate that integrated motion metrics from FIDnavs embedded in structural MRI are a useful predictor of diagnostic image quality, which translates to substantial time and cost savings when applied to pediatric MRI examinations.

## 1 INTRODUCTION

Subject motion is a substantial problem for the acquisition of high‐quality MRI data in children and other uncooperative patient populations.^1^, ^2^ Resulting artifacts including ghosting, blurring, and signal dropout can significantly degrade image quality,^3^ necessitating repeat scans^4^ and the widespread use of sedation and general anesthesia.^5^ These practices dramatically increase the time and cost involved in acquiring diagnostically useful images. The prevalence of repeated sequences due to motion artifacts has been estimated at around 20%, yielding additional costs in excess of $115 000 per scanner, per year,^4^ totaling $1.4 billion yearly in the United States alone.^6^ Another study estimated the annual costs to hospitals arising from patient head motion at $45 066, which increased to $364 242 when including pediatric scans where anesthesia was deemed necessary to avoid motion.^7^ Approximately a quarter of all pediatric exams are performed with monitored anesthesia care, with the reported rate rising up to 80% in children aged 1‐6 years.^5^ Sedation and general anesthesia result in significantly longer visit durations compared to awake patients, with reported visit costs up to 3 and 10 times higher, respectively.^8^ Furthermore, sedation is not always successful at mitigating patient motion^9^ and, given the potential for adverse short‐term and long‐term consequences,^10^ its use should be weighed against the potential benefit and, therefore, must be carefully justified in research settings.

Research has shown that behavioral interventions, including watching a movie and receiving real‐time feedback about motion levels, can help mitigate head movement in children during functional MRI studies.^11^ However, there is currently no tool or device on the market that has achieved widespread acceptance to provide motion monitoring during conventional clinical MRI sequences. Optical^12^, ^13^ and RF‐based^14^, ^15^ tracking systems can provide highly accurate, real‐time motion estimates, and suspending data acquisition during periods of head motion measured by optical tracking has been shown to be successful in improving image quality.^16^ Ideally a bite‐bar should be used to rigidly couple motion of the tracked marker to the skull; however, this is not feasible for pediatric studies. Instead, markers are typically placed on the forehead or bridge of the nose, making them highly susceptible to non‐rigid pseudo‐motion, which may lead to false positive motion events and unnecessarily prolonged scan times. MR‐based navigators have the advantage of providing intrinsic tracking information (without any additional hardware requirements), either from the acquired *k*‐space data itself^17^, ^18^ or from additional navigator echoes inserted into the sequence.^19^, ^20^, ^21^ Several studies have demonstrated the ability of image‐based prospective motion navigation^22^ to improve both diagnostic image quality and cortical morphometry measurements in adult and pediatric MRI scans,^23^, ^24^ particularly when combined with prospective data reacquisition.^25^ However, image‐based navigators require intrinsic “dead time” to be present in the host sequence to achieve sufficient tracking accuracy without incurring a substantial time penalty.

FID navigators (FIDnavs) measure signal from the receiver coils without any gradient encoding, meaning they can be acquired extremely rapidly with minimal impact on the magnetization or overall scan time. These ultra‐short navigator signals are sensitive to head motion due to the localized spatial sensitivities of each coil in a multi‐channel receiver array.^26^ FIDnavs have been applied to detect rigid and non‐rigid motion events in a variety of other applications, including carotid^27^ and abdominal^28^ imaging, and diffusion‐weighted brain MRI.^29^ Detecting motion with FIDnavs typically involves combining FIDnav signals from multiple channels into a single measurement, which facilitates use of a simple, empirical threshold to detect motion. A variety of FIDnav motion detection algorithms have been proposed; however, there is no consensus on the optimal metric or threshold for reliable detection of motion related to image degradation. Previous studies have demonstrated the utility of FIDnav motion detection in volunteer scans with deliberate, choreographed head movements,^26^, ^27^, ^28^, ^29^, ^30^ but there has been a lack of validation in realistic clinical scenarios with involuntary patient motion. The purpose of this study was to investigate the ability of FIDnav motion detection to prospectively predict diagnostic utility. The sensitivity and specificity of four FIDnav detection algorithms were evaluated relative to radiologic evaluation of image quality in a pediatric patient cohort. The potential time and cost savings arising from FID‐navigated motion monitoring were also estimated.

## 2 METHODS

### 2.1 MRI data acquisition

**FIGURE 1:**
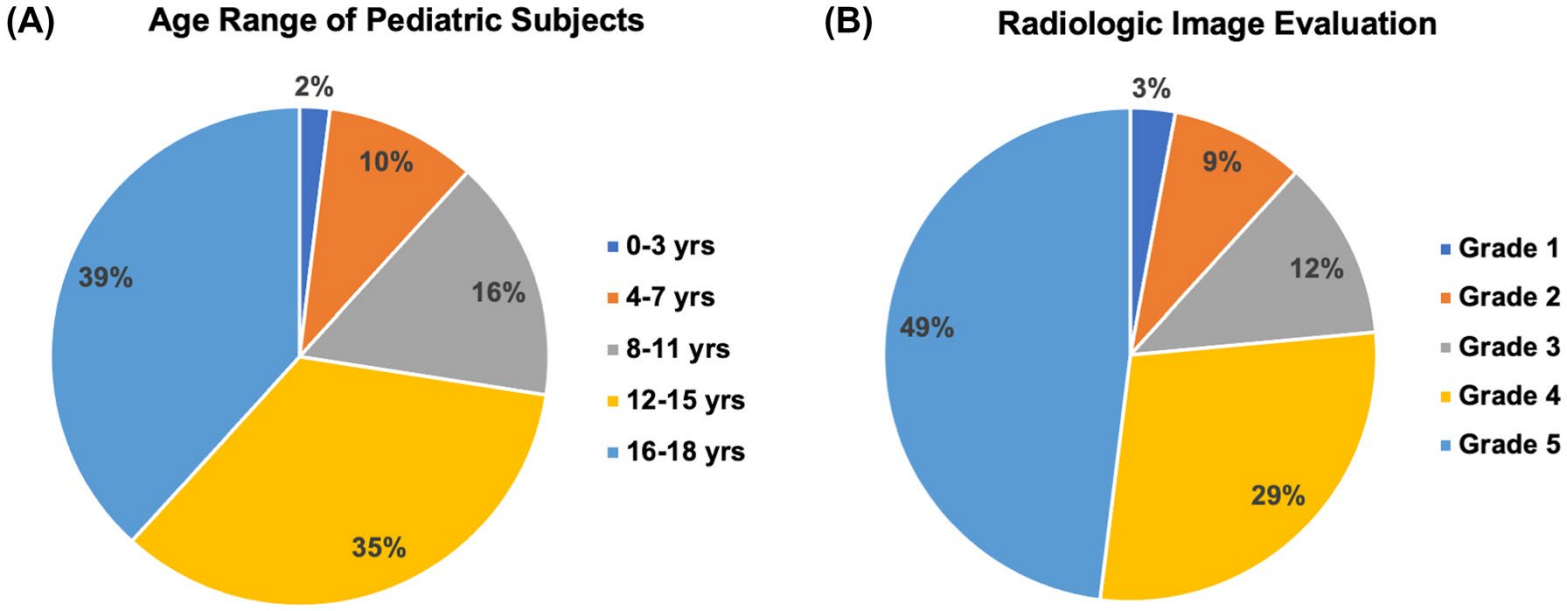
Distribution of ages of pediatric patients (A) and radiologic evaluation of image grades (B)

A total of 102 pediatric patients (49 female) were scanned between October 2017 and August 2019 at our hospital's outpatient facility as part of a clinical quality improvement study. The study of previously acquired data was carried out under a research protocol approved by the local Institutional Review Board (IRB) with a waiver of informed consent. Patients scanned without sedation at 3T with an FID‐navigated MPRAGE sequence were selected for inclusion in this study. Patients’ ages ranged from 0 to 18 years, with median age 14 years; a detailed breakdown of patient age range is shown in Figure 1A. Eight adult patients (age 19‐35) were also scanned to provide a baseline for k‐space weighting.

All examinations were performed on a 3T MR scanner (MAGNETOM Trio, A Tim System; Siemens, Erlangen, Germany) using the vendor‐supplied 32‐channel head coil. A T_1_‐weighted MPRAGE sequence was acquired for each patient with the following scan parameters: repetition time (TR) 1540 ms, inversion time (TI) 800 ms, echo time (TE) 2.47 ms, flip angle 9°, receiver bandwidth 200 Hz/pix, field of view (FOV) 220 × 220 × 152 mm, resolution 0.9 mm isotropic, GRAPPA acceleration factor 2, total acquisition time 4.2 minutes. An FID navigator module, comprising an ADC readout of 0.2 ms duration, was embedded in the sequence following each radiofrequency (RF) excitation pulse, prior to image readout. This increased the minimum echo time for the MPRAGE sequence by 0.2 ms The overall acquisition time for the sequence was not affected as the dead time in an MPRAGE scan is sufficiently large. A Cartesian sampling trajectory was used with the center of the phase encoding direction acquired halfway through the scan. Acquisitions were non‐selective and acquired in the sagittal plane. Sequence parameters were chosen to closely match those typically used in clinical studies. N = 26 of 102 scans were acquired with “fast” water excitation (two binomial pulses) as per the clinical protocol. N = 12 of 102 scans were acquired post‐contrast. Foam padding was used within the head coil to restrict motion, as is done in clinical practice. No specific instructions were given to subjects, other than to remain as still as possible for the duration of the scan.

### 2.2 Image quality scoring

Scans were graded by a radiologist (C.J., 4 years of experience) based on the severity of motion artifacts and diagnostic image quality. Images were ranked using a five‐point scale as follows: (a) severe motion artifact, non‐diagnostic image without anatomic information (eg, gross anatomic distortion such as hydrocephalus or a large tumor would be obscured); (b) severe motion artifact, non‐diagnostic image with limited gross anatomic information (eg, ventricular size, midline shift); (c) moderate motion artifact, diagnostic quality is compromised but some information is still obtained; (d) mild motion artifact, exam remains fully diagnostic; and (e) no appreciable motion artifact. A subset of scans (N = 55) were also graded by a second radiologist (J.R., 3 years of experience) to establish inter‐rater reliability.

### 2.3 FIDnav motion detection

The raw FID signal measured by each receiver channel consisted of 64 complex data points sampled in 0.2 ms after each RF excitation pulse in the gradient echo readout train. The middle 32 samples were averaged to remove effects due to the electronic adjustments of the ADC.^26^ FIDnav samples from all readouts in each echo train were further averaged to yield a single complex navigator signal per channel (FIDnav signal vector) for each TR. The first three time points were discarded to allow the signal to reach steady state. To compress the multi‐channel FIDnav data into a single global motion metric per TR, individual coil measurements were combined using the following algorithms:

1. Normalized mean absolute change in FIDnav over all channels relative to the reference FIDnav signal ($FIDnav_{\Delta ref}$)

(1) $$FIDnav_{\Delta ref}\;\left(i\right)=\frac{1}{N_{c}}\sum_{j=1}^{N_{c}}\frac{∥s_{j}\left(i\right)\left|‐\right|s_{j}\left(ref\right)∥}{\left|s_{j}\left(ref\right)\right|}$$

where $s_{j}\left(i\right)$ is the complex FIDnav signal measured from the *j*th coil at the *i*th time point (TR) where *i* is in the range 2 … *N_TR_*, $s_{j}\left(\mathrm{ref}\right)$ is the reference FIDnav signal averaged from the first three time points, $\left|\cdot \right|$ denotes the magnitude operator and *N_c_* is the number of channels in the coil array. This is based on the algorithm originally proposed by Kober et al^26^ and subsequently used for detecting swallowing motion in carotid MRI.^27^

- Normalized mean absolute change in FIDnav over all channels relative to the previous TR ($FIDnav_{\Delta }$)

(2) $$FIDnav_{\Delta }\left(i\right)=\frac{1}{N_{c}}\sum_{j=1}^{N_{c}}\frac{\left|\left|s_{j}\left(i\right)\right|‐\left|s_{j}\left(i‐1\right)\right|\right|}{\left|s_{j}\left(i‐1\right)\right|}$$

This metric is similar to Equation (1), but motion is detected relative to the previous repetition, rather than an initial reference time point. This was chosen as subjects generally do not return to their initial position following a motion event.

- Normalized mean change in absolute FIDnav over the three channels with maximal signal change at each time point ($FIDnav_{\Delta \max}$)

(3) $$FIDnav_{\Delta \max}\left(i\right)=\frac{1}{3}\sum_{j=1}^{3}\frac{\left|\left|s_{j}\left(i\right)\right|‐\left|s_{j}\left(i‐1\right)\right|\right|}{\left|s_{j}\left(i‐1\right)\right|}$$

This metric was previously proposed to increase sensitivity to fast motion by only averaging data from channels exhibiting maximum signal change.^30^ Averaging over the maximally changing three channels should minimize the effect of random signal fluctuations, while maximizing motion‐induced signal changes.

- Cross correlation coefficient (CCC) between absolute FIDnav signal projection vectors ($FIDnav_{\mathrm{CCC}}$)

(4) $$FIDnav_{\mathrm{CCC}}\left(i\right)=1‐\frac{\sum_{j=1}^{N_{c}}\left[\left(s_{j}\left(i\right)‐\bar{s_{j}\left(i\right)}\right)\left(s_{j}\left(i‐1\right)‐\bar{s_{j}\left(i‐1\right)}\right)\right]}{\sqrt{\frac{1}{N_{c}‐1}\sum_{j=1}^{N_{c}}{\left(s_{j}\left(i\right)‐\bar{s_{j}\left(i\right)}\right)}^{2}}\sqrt{\frac{1}{N_{c}‐1}\sum_{j=1}^{N_{c}}{\left(s_{j}\left(i‐1\right)‐\bar{s_{j}\left(i‐1\right)}\right)}^{2}}}$$

where $\bar{s_{j}\left(i\right)}$ denotes the mean of $s_{j}\left(i\right)$ and $\sum_{j=1}^{N_{c}}{\left(s_{j}\left(i\right)‐\bar{s_{j}\left(i\right)}\right)}^{2}$ denotes the variance of $s_{j}\left(i\right)$. This algorithm was originally applied for FIDnav‐based detection of abdominal motion.^28^ A change in CCC relative to the previous TR implies that the load distribution of the coil elements has changed, indicating a motion event.

### 2.4 Prediction of diagnostic image quality

To evaluate the ability of FIDnavs to predict motion affecting diagnostic image quality, these four global FIDnav metrics were further compressed into single measures of the total impact of motion occurring during the scan as follows:

- Integrated FIDnav motion score over time (FIDnav):

(5) $$FIDnav=\frac{1}{n\cdot TR}\sum_{i=1}^{n‐1}FIDnav_{i}$$

This represents the numerical integration of the total FIDnav motion score, where $FIDnav_{i}$ is a placeholder for the four metrics used and *n* is the total number of phase‐encoding steps in the acquisition.

- Partition‐weighted integrated FIDnav motion score (wFIDnav):

(6) $$wFIDnav=\frac{1}{n\cdot TR}\sum_{i=1}^{n}w_{i}\cdot FIDnav_{i}$$

where $w_{i}$ represents the 1D weighting associated with the *i*th phase‐encoding step. This metric reflects the fact that *k*‐space energy; hence, the impact of motion occurring at each encoding step is non‐uniformly distributed along the phase‐encoding direction.^3^ Weightings were calculated from the norm of each acquired *k*‐space plane,^31^ averaged across the eight co‐operative adult subjects scanned with the same sequence parameters. The weighting function used to compute wFIDnav is plotted in Supporting Information Figure S1, which is available online.

Spearman rank correlation was evaluated to determine whether these aggregate FIDnav‐based motion metrics correlated with radiologic evaluation grade. *P* values were calculated to determine if group‐wise differences were statistically significant.

A receiver operating characteristic (ROC) analysis was performed to compare the sensitivity and specificity of each FIDnav‐based metric to detect differences between non‐diagnostic images (Grades 1‐2) and images with some diagnostic value (Grades 3‐5), as well as between images with impaired diagnostic quality (Grades 1‐3) and fully diagnostic images (Grades 4‐5). Sensitivity (*SE*) and specificity (*SP*) were defined from the ratio of true and false positives (*TP*, *FP*) and true and false negatives (*TN*, *FN*) as follows:

(7) $$\begin{array}{c} SE=\frac{\mathrm{TP}}{TP+FN}\\ SP=\frac{\mathrm{TN}}{TN+FP} \end{array}$$

**FIGURE 2:**
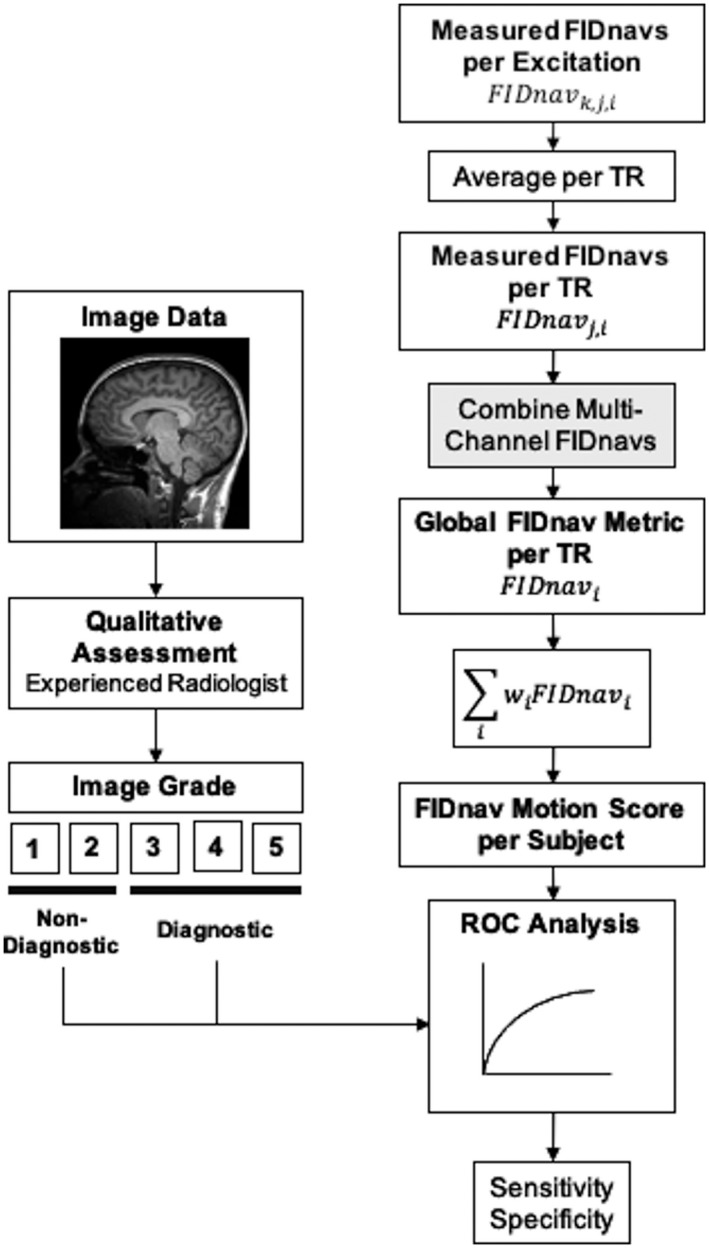
Algorithm for evaluating prediction of diagnostic image quality using FID navigators. Measured multi‐channel FIDnav signals from each excitation *k* are averaged to obtain a FIDnav measurement per channel *j* and TR *i*. Multi‐channel FIDnavs are combined using four different algorithms to create a global FIDnav motion metric per TR, and then, integrated to generate a FIDnav motion score for each subject. Images are evaluated as diagnostic or non‐diagnostic based on expert radiologic evaluation, and a ROC analysis is conducted across all subjects to determine the sensitivity and specificity of FIDnav motion detection

A positive case refers to a detected motion‐corrupted scan that should be aborted and required, and a negative case refers to a detected diagnostic scan where the motion threshold is not exceeded. Youden's index, defined as: $J=SE_{c}+SP_{c}‐1$ was calculated for each threshold *c*.^32^ The optimal empirical threshold for detection of image quality degradation was determined as the value that maximizes this index. A summary of the algorithm used for motion detection is shown in Figure 2.

To provide an estimate of the detection accuracy for unseen datasets, bootstrap datasets were constructed by randomly drawing subjects with replacement to create samples of size N = 102. This process was repeated *b* = 1000 times and the optimal threshold was computed from a ROC analysis of each set of bootstrap samples. Sensitivity and specificity were estimated from subjects not contained in each training dataset (“out‐of‐bag” samples). This leave‐one‐out bootstrapping approach solves the problem with over‐fitting that occurs when the test data is contained within the training data, but is still biased due to non‐distinct observations in the bootstrap samples that result from sampling with replacement. The 0.632 estimator was applied to solve this bias problem as proposed by Efron et al^33^:

(8) $$Acc^{\text{boot}}=\frac{1}{b}\sum_{i=1}^{b}0.368\cdot Acc_{r,i}+0.632\cdot Acc_{h,i}$$

where $Acc_{r,i}$ is the resubstitution accuracy for the *i*th bootstrap sample and $Acc_{h,i}$ is the accuracy of the out‐of‐bag sample.

### 2.5 Potential time and cost savings with FIDnav motion monitoring

The optimal thresholds from the ROC analysis were retrospectively applied to the FIDnav motion metrics to determine the potential time savings that could be realized by terminating the acquisition of motion‐corrupted scans at the point when the FIDnav‐based metrics exceeded this threshold. For this analysis, it was assumed that all images ranked in Grades 1‐2 would be reacquired following completion of the non‐diagnostic scan, giving a total acquisition time of 2*TA*, whilst scans with acceptable or good diagnostic quality (Grades 3‐5) would not be repeated. The overall time savings are based on the detection accuracy of true positives (detected non‐diagnostic scans) and false positives (diagnostic scans falsely classified as motion‐corrupted). For any scan of non‐diagnostic image quality, if the integrated motion metric exceeded the predefined threshold value (computed from ROC analysis), the fraction of the time remaining (*FR*) was recorded, as this represents the time saved in acquiring non‐diagnostic image data. Thus, the total time spent scanning subjects with non‐diagnostic images was: $TA\left(2‐FR\right)$. For any scan of diagnostic quality, if the integrated motion metric exceeded the threshold value, the fraction acquired (*FA*) was recorded, as this represents time wasted unnecessarily restarting the scan. Thus, the total time spent scanning subjects with diagnostic images was: $TA\left(FA+1\right)$. In cases where no motion is detected (true negatives and false negatives), there are no time savings or penalties associated with FIDnav motion monitoring. The aggregate time savings were computed across all subjects and expressed as a percentage of the total scan time:

(9) $$T_{\text{Savings}}\left(\%\right)=100\cdot \left\lceil \frac{\sum_{i=1}^{\mathrm{nTP}}FR_{i}‐\sum_{j=1}^{\mathrm{nFP}}\left(FA_{j}\right)}{2\left(nTP+nFN\right)+nTN+nFP}\right\rceil$$

where *nTP, nFP, nTN*, and *nFN* represent the number of true positives, false positives, true negatives, and false negatives, respectively. The optimal threshold was also calculated with respect to directly maximizing the time savings across all scans.

The financial costs of reacquiring motion‐corrupted data in this study and potential savings from FID‐navigated motion monitoring were also estimated. These calculations were based on the cost of a standard brain MRI (without contrast or anesthesia) at our institution, which is $2828, assuming a 45‐min time slot. The additional cost of repeating motion‐corrupted MPRAGE scans (Grades 1‐2) was estimated, given a 4.2 minutes scan duration. The aggregate cost savings from FID‐navigated motion monitoring during non‐diagnostic scans (FR), as well as additional costs associated with unnecessarily reacquiring diagnostic image data (FA) was computed to yield an estimate of the total cost savings for the patients in this study.

## 3 RESULTS

**FIGURE 3:**
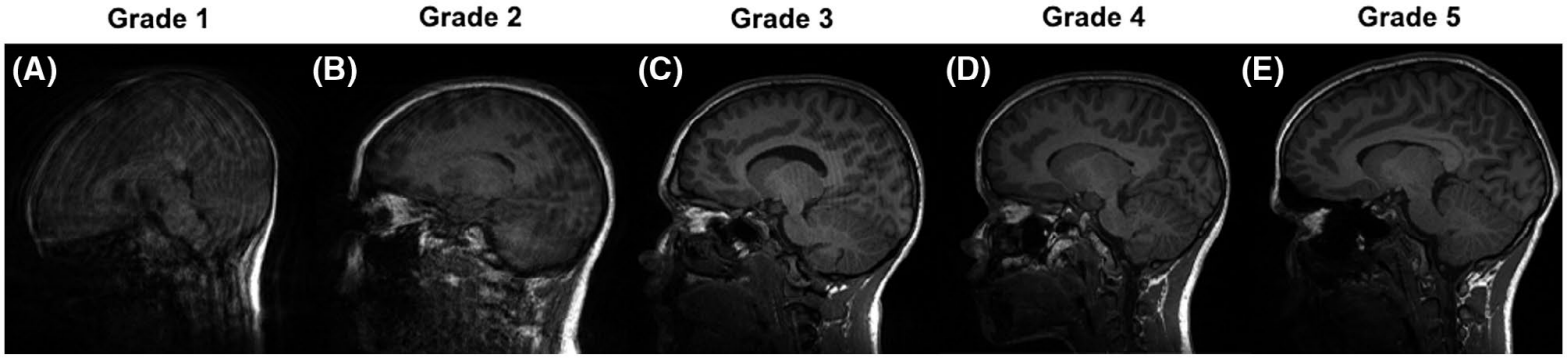
Sagittal MPRAGE images acquired in pediatric patients, representative of each motion grade, as evaluated by an expert radiologist. Image grade increases from left to right. Severe ghosting, blurring, and ringing artifacts obscuring anatomical information are present in images ranked as Grades 1 (A) and 2 (B). C, A moderate ringing artifact is observed. Images ranked as Grades 4‐5 are considered fully diagnostic; there are subtle arcs from ghosting artifacts present in (D), while there are no appreciable artifacts in (E)

Images were ranked by two experienced radiologists on a five‐point scale to facilitate correlation of image quality with FIDnav motion metrics. The distribution of image quality scores is shown in Figure 1B. The presence of severe motion artifacts meant that 12% of all scans were non‐diagnostic (Grades 1‐2). A further 12% had compromised diagnostic value due to motion (Grade 3). There was excellent agreement between the two raters’ evaluation of image quality (Cohen's weighted κ = 0.86, 95% CI [0.68‐1.0], *P* < .001). All disagreements between raters occurred for scans with quality ratings in adjacent categories. Representative images corresponding to each motion grade are shown in Figure 3.

### 3.1 Prediction of diagnostic image quality

**FIGURE 4:**
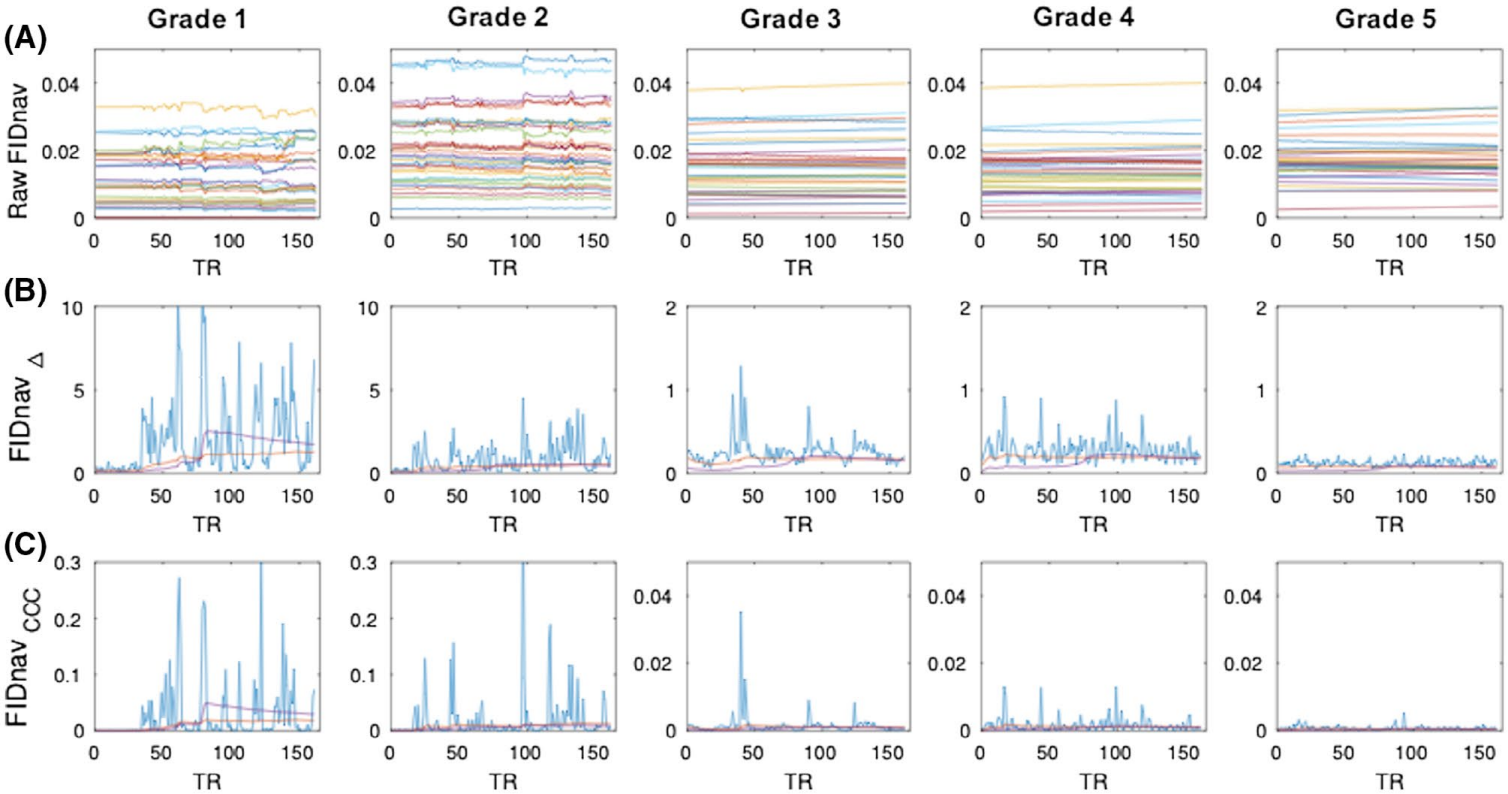
(A) Multi‐channel FIDnav signal traces corresponding to images acquired with each motion grade. Computation of the motion metrics $FIDnav_{\Delta }$ (B) and $FIDnav_{\mathrm{CCC}}$ (C) from the multi‐channel FIDnav data (blue line), and the corresponding integrated (red) and partition‐weighted integrated (purple) motion scores (shown in in mm s^−1^)

**FIGURE 5:**
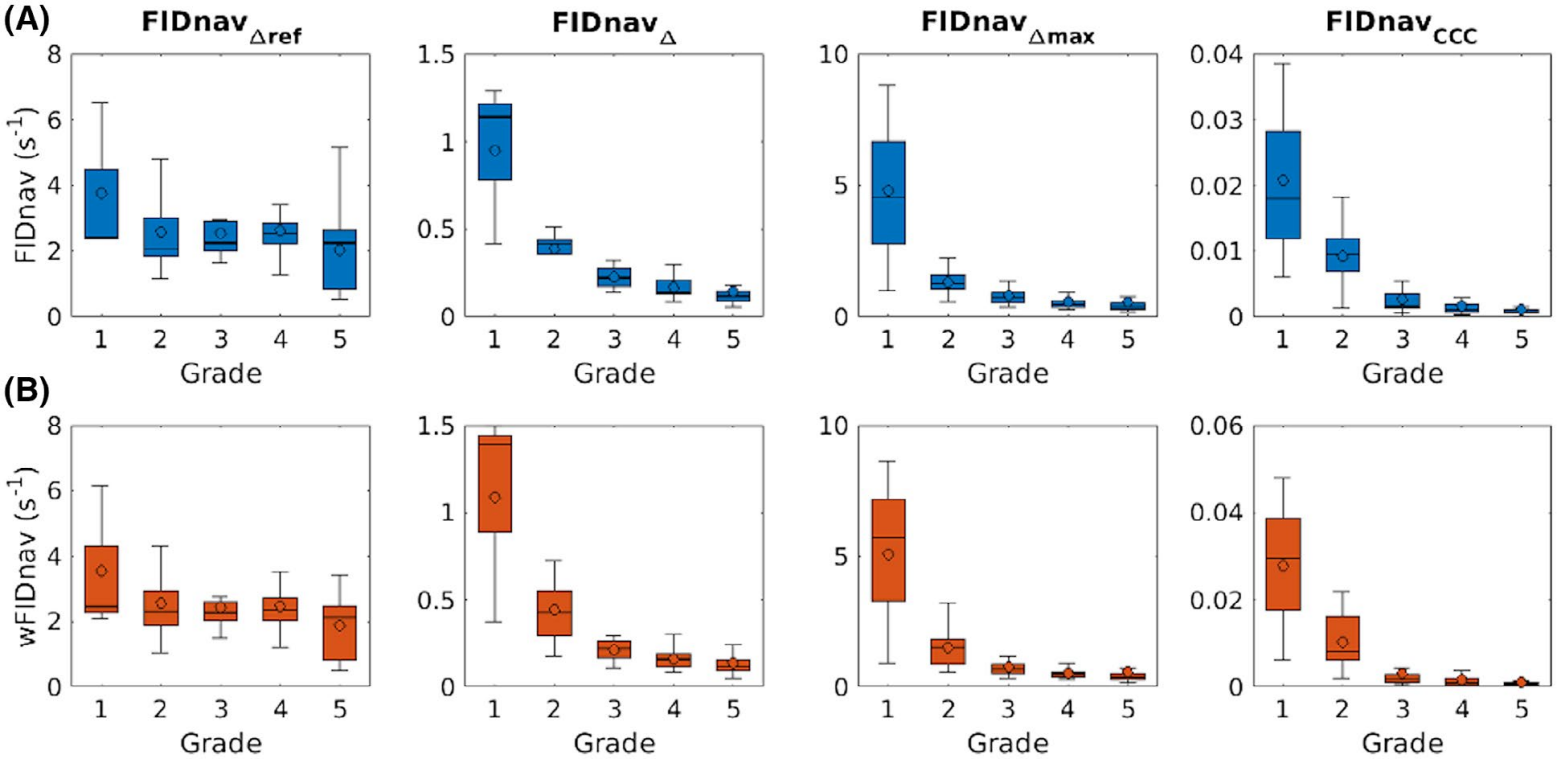
Boxplots showing the distribution of integrated FIDnav metrics (A) and partition‐weighted integrated FIDnav metrics (B) corresponding to each image grade, as evaluated by a radiologist

Figure 4 shows the multi‐channel FIDnav signal traces corresponding to the images from Grades 1‐5 displayed in Figure 3. The temporal resolution of FIDnav motion measurement is determined by the TR of the sequence (1.54 seconds in this study). The derived metrics $FIDnav_{\Delta }$ and $FIDnav_{\mathrm{CCC}}$ are shown, alongside the integrated and partition‐weighted integrated motion scores for each subject. The relationship between aggregate FIDnav motion scores and radiologic evaluation of image quality is summarized in Figure 5. The mean and standard deviation of FIDnav metrics corresponding to each motion grade is displayed in Table 1. All metrics apart from $FIDnav_{\Delta ref}$ were significantly correlated with image grade (Spearman rank correlation coefficient 0.56‐0.60; *P* < .001). Weighting the motion metrics by an estimate of the *k*‐space signal at each time point led to a slight reduction in the correlation with expert‐rated image quality. Thus, only the integrated FIDnav motion metrics (without *k*‐space partition weighting) are considered in the following analysis.

**TABLE 1:** Summary integrated FIDnav statistics (mean ± SD) for each image grade derived from each FIDnav metric

	FIDnav (s^−1^)				wFIDnav (s^−1^)			
	$FIDnav_{\Delta ref}$	$FIDnav_{\Delta }$	$FIDnav_{\Delta \text{max}}$	$FIDnav_{\mathrm{CCC}}*100$	$FIDnav_{\Delta ref}$	$FIDnav_{\Delta }$	$FIDnav_{\Delta \max}$	$FIDnav_{\mathrm{CCC}}*100$
Grade 1 (n = 3)	3.77 ± 2.37	0.95 ± 0.47	4.79 ± 3.90	2.09 ± 1.65	3.56 ± 2.25	1.09 ± 0.62	5.08 ± 3.90	2.78 ± 2.10
Grade 2 (n = 9)	2.56 ± 1.17	0.39 ± 0.12	1.30 ± 0.451	0.92 ± 0.50	2.54 ± 1.08	0.44 ± 0.18	1.51 ± 0.81	1.04 ± 0.70
Grade 3 (n = 12)	2.56 ± 0.90	0.22 ± 0.06	0.81 ± 0.41	0.26 ± 0.21	2.42 ± 0.76	0.21 ± 0.06	0.78 ± 0.48	0.31 ± 0.46
Grade 4 (n = 29)	2.68 ± 1.23	0.17 ± 0.06	0.55 ± 0.24	0.16 ± 0.16	2.49 ± 1.11	0.16 ± 0.06	0.52 ± 0.24	0.16 ± 0.15
Grade 5 (n = 49)	2.01 ± 1.07	0.14 ± 0.09	0.58 ± 0.87	0.10 ± 0.09	1.90 ± 1.07	0.14 ± 0.10	0.55 ± 0.77	0.10 ± 0.09
Spearman Correlation	0.22	0.60	0.56	0.57	0.25	0.59	0.54	0.55

### 3.2 Sensitivity and specificity of FIDnav motion detection

**FIGURE 6:**
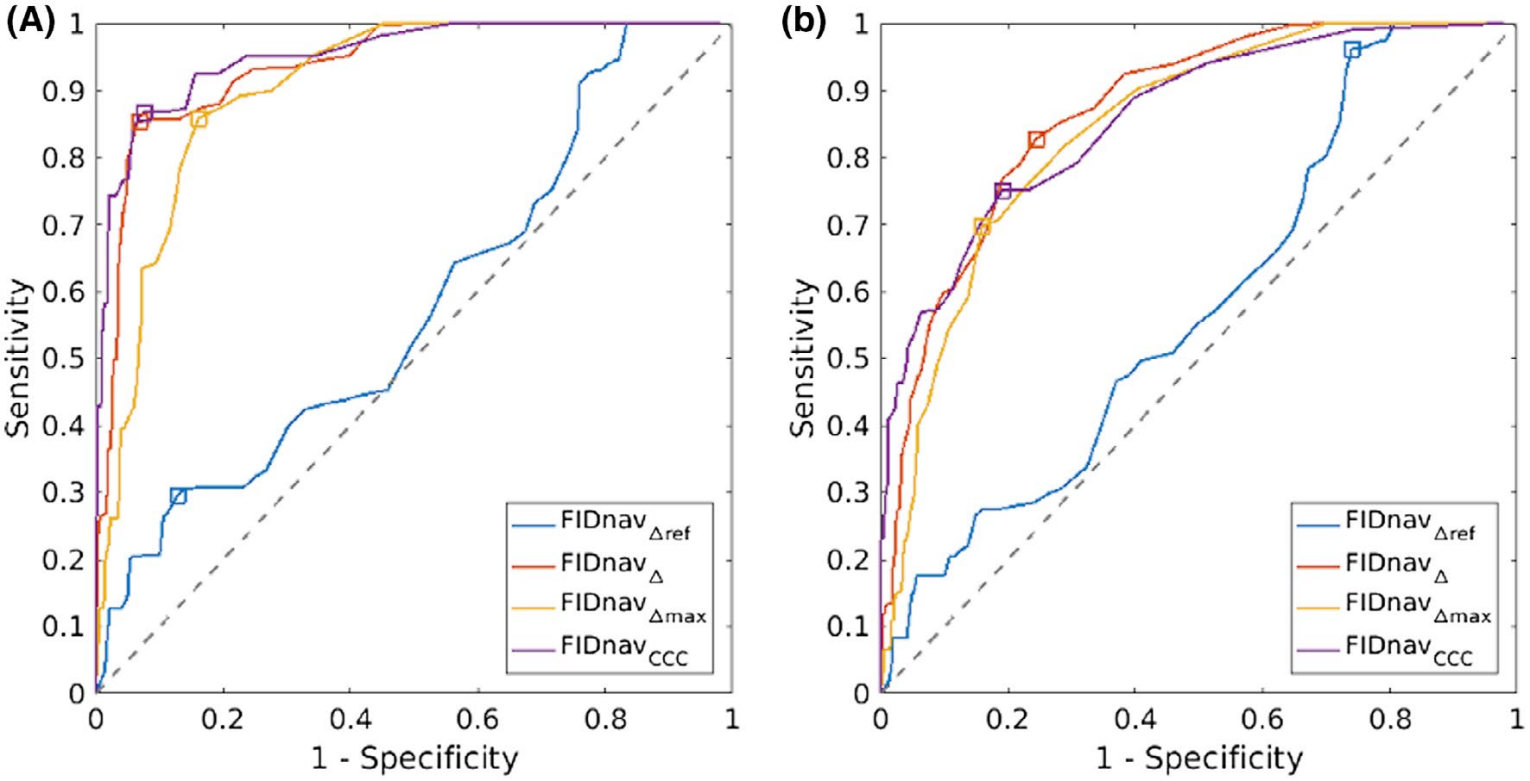
ROC curves averaged over bootstrap training datasets and operating points for each integrated FIDnav metric showing sensitivity and specificity for detecting the difference in motion corruption between image Grades 1‐2 and 3‐5 (severe – moderate motion artifacts; A) and Grades 1‐3 and 4‐5 (moderate – mild motion artifacts; B)

The results of the ROC analysis for all four integrated FIDnav motion metrics are summarized in Table 2. Computing the cross‐correlation coefficient between FIDnav signal vectors ($FIDnav_{\mathrm{CCC}}$) achieved the highest combined sensitivity and specificity (*J* = 0.86 ± 0.08) for detecting non‐diagnostic images (Figure 6A), outperforming computing the percentage signal change across all or maximally changing channels. A sensitivity of 0.85 for $FIDnav_{\mathrm{CCC}}$ means that 85% of non‐diagnostic images were correctly identified, while a specificity of 0.93 means that 7% of diagnostic images were incorrectly flagged as non‐diagnostic. The FIDnav‐based metrics were less sensitive in detecting images with impaired diagnostic quality (Grades 1‐3 vs. 4‐5), with sensitivity 0.73 and specificity 0.80 for $FIDnav_{\mathrm{CCC}}$ (Figure 6B).

**TABLE 2:** ROC analysis area under the curve and Youden's index with mean and SD estimated via bootstrapping, optimal threshold, and sensitivity and specificity using the 0.632 estimator for each integrated FIDnav motion score (computed without partition weighting)

Integrated motion score	Grades 1‐2 vs. 3‐5					Grades 1‐3 vs. 4‐5				
	AUC	J	Th	SE	SP	AUC	J	Th	SE	SP
$FIDnav_{\Delta ref}$	0.55 ± 0.09	0.28 ± 0.10	1.48	0.54	0.58	0.55 ± 0.07	0.27 ± 0.06	1.09	0.80	0.39
$FIDnav_{\Delta }$	0.93 ± 0.03	0.83 ± 0.09	0.18	0.82	0.92	0.86 ± 0.04	0.66 ± 0.08	0.11	0.79	0.78
$FIDnav_{\Delta \max}$	0.91 ± 0.03	0.77 ± 0.08	0.51	0.84	0.84	0.84 ± 0.04	0.63 ± 0.08	0.37	0.76	0.78
$FIDnav_{\mathrm{CCC}}$	0.94 ± 0.03	0.86 ± 0.08	0.0026	0.85	0.93	0.85 ± 0.04	0.62 ± 0.08	0.0013	0.73	0.80

Abbreviations: AUC, area under the operating curve; J, Youden's index; SE, sensitivity; SP, specificity; Th, optimal threshold.

### 3.3 Potential time and cost savings with FIDnav motion monitoring

**FIGURE 7:**
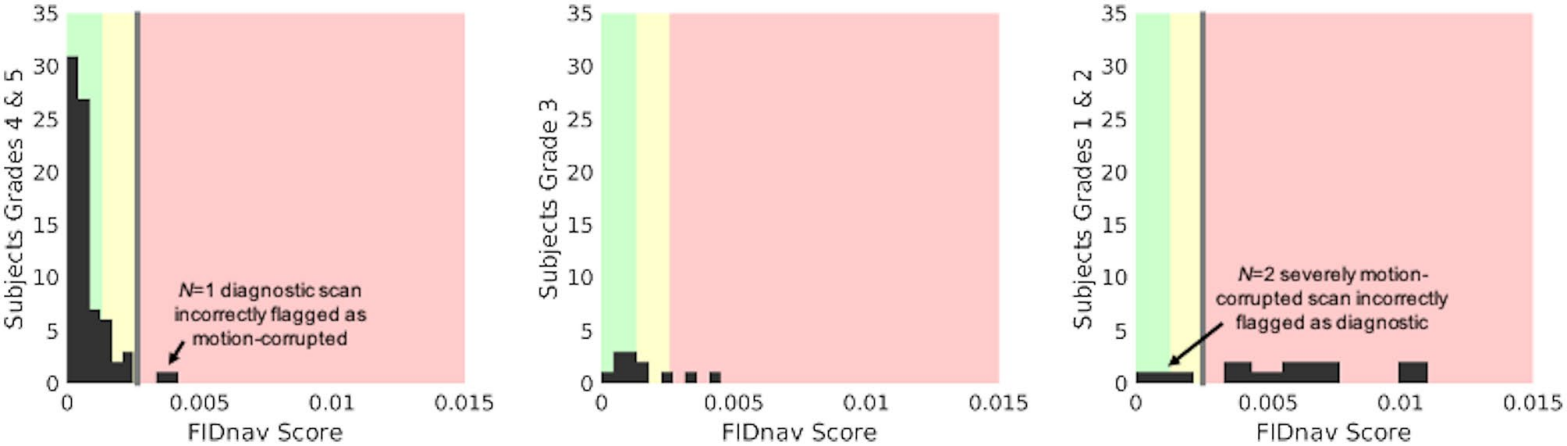
Histograms showing proportion of scans correctly flagged as diagnostic or non‐diagnostic using thresholds derived from the ROC analysis for the $FIDnav_{\mathrm{CCC}}$ metric. Green, yellow, and red regions indicate images predicted to lie within Grades 4‐5, Grade 3, and Grades 1‐2, respectively, according to the optimal FIDnav thresholds

The integrated cross‐correlation between FIDnav signal vectors (*FIDnav_CCC_*) was used to compute the potential time and cost savings that may be realized using FIDnav motion monitoring to detect non‐diagnostic images as this metric was shown to have the highest overall detection power. Histograms of this integrated FIDnav motion score are shown in Figure 7 for subjects in Grades 1‐2 (non‐diagnostic), Grade 3 (some diagnostic value), and Grades 4‐5 (fully diagnostic), alongside the optimal thresholds for detecting motion‐corrupted scans. Applying the optimal threshold to the $FIDnav_{\mathrm{CCC}}$ metric to detect non‐diagnostic images yielded total time savings of 6.8% across all scans (N = 102); only 1.1% of the overall imaging time was ”wasted” reacquiring diagnostic quality images. The FR for detected non‐diagnostic scans was 0.82 ± 0.18 (mean ± SD), while the FA for falsely detected diagnostic scans was 0.13 ± 0.14. Using the optimal threshold with respect to maximizing time efficiency yielded overall time savings of 7.1%.

Based on a 4.2 minutes MPRAGE scan, and assuming all scans ranked as Grades 1‐2 would need to be reacquired, the additional cost of repeat sequences due to motion artifacts in this study was estimated at $3167, for 50 minutes of additional scan time. The aggregate time savings from FIDnav motion monitoring during non‐diagnostic scans was estimated at 39 minutes, while the time wasted in reacquiring diagnostic images was estimated at 5 minutes. This yields a total cost saving of $2080 for the 102 patients scanned in this study. Assuming eight MPRAGE scans are run on each scanner per day, this would correspond to savings of approximately $54 k per scanner per year.

## 4 DISCUSSION

Despite substantial technological advances in the past few decades, head motion remains a huge problem for successful MRI in unsedated children. A large proportion of the available scan time can be wasted repeatedly acquiring non‐diagnostic images corrupted by motion, often without any indication prior to the final image reconstruction. An ideal motion detection algorithm should have high sensitivity and specificity to identify head motion adversely affecting data quality, without unnecessarily prolonging the scan time required for reacquisition of diagnostic‐quality scans. The aim of this work was to investigate the ability of FID navigator‐based motion metrics to determine clinically relevant levels of motion in pediatric patients scanned in a realistic clinical setting. Our results show that integrated motion metrics from FIDnavs embedded in a structural MPRAGE sequence are correlated with expert radiologic evaluation of image quality. Integrating the cross‐correlation between FIDnav signal vectors (*FIDnav_CCC_*) had the highest power for detecting non‐diagnostic images. Substantial time and cost savings could be realized by applying a cut‐off threshold to this integrated FIDnav metric to inform termination of non‐diagnostic acquisitions prior to completion.

In this study, scans ranked as Grade 4‐5 were judged to be fully diagnostic. Scans ranked as Grade 3 were corrupted by motion to a degree that they still retained some diagnostic value. The need to repeat these scans would likely depend on both the clinical context and quality of other images in the series. Scans ranked as Grades 1‐2 were severely corrupted by motion and deemed to have no clinical value. The results of the current study indicate that FIDnavs are particularly useful in flagging non‐diagnostic images with more severe motion artifacts, which would need to be repeated regardless of clinical indication. FID‐navigated motion monitoring had moderate sensitivity and specificity when Grade 3 images were also classified as non‐diagnostic. The optimal threshold is ultimately dependent on the study context and is a trade‐off between sensitivity and specificity, and the ability to detect more subtle motion artifacts.

### 4.1 Impact of motion on diagnostic image quality

The impact of motion on image quality in MRI is a subject of ongoing research. The nature and severity of motion artifacts are dependent on both the amplitude and timing of the motion relative to the encoding of the central *k*‐space lines, which contain the bulk image contrast, as well as the exact pulse sequence parameters and reconstruction method.^3^ A previous study investigating the effect of motion on MRI quality in a large pediatric patient cohort found that diagnostic image quality was highly correlated with the mean displacement and ratio of motion‐free time relative to a reference time point corresponding to acquisition of the *k*‐space center.^1^ Another study found that accounting for both the amplitude of the signal acquired at the time of motion, as well as measured head speed was important in predicting the impact of head motion on image quality.^16^

In this work, weighting the integrated motion score by the energy contained within the acquired *k*‐space data led to a slight reduction in the correlation between FIDnav motion score and image grade. This is likely due to the nature of pediatric head motion: subjects who are prone to motion tend to move continuously throughout the scan, ie, the relative timing of errors in k‐space encoding is overwhelmed by the amount of error occurring over the course of the scan. If subjects performed a smaller number of abrupt head motions, similar to the study design in,^16^ then, the timing of these events in k‐space would become more important; however, this is uncharacteristic of how pediatric subjects move in practice.

### 4.2 Time and cost savings

Due to the expense of providing MRI services, even modest improvements in efficiency and utilization can dramatically reduce excess costs.^34^, ^35^ The cost savings analysis performed in this study is just considering motion monitoring during the MPRAGE sequence. Extending FID‐navigated motion monitoring capabilities to other sequences is expected to further increase potential savings. This simple approximation also does not consider the additional delays associated with decision‐making by the technologist and radiologist following acquisition of a motion‐corrupted scan, nor does it consider the need to potentially recall patients from home for a second scan if motion is not detected, or the additional costs when a repeat scan with anesthesia is necessary to mitigate motion artifacts.^7^

The time savings analysis assumes that the second scan following intervention by the technologist is successful and free of motion artifacts. While in some cases, patient motion may increase throughout the scan due to increasing fatigue and discomfort, in our experience, once the technologist is aware the subject is moving they can often suggest interventions to make the patient more comfortable and/or motivate the patient to hold still for the following scan.

### 4.3 Advantages and limitations

FIDnavs are an attractive approach for pediatric head motion monitoring as they can be acquired with high temporal resolution in virtually any sequence, with minimal time penalty, and do not require any additional hardware or fiducial markers. Combining FIDnav data from multiple channels into a single motion metric enables real‐time motion monitoring and the use of an empirical threshold to detect different levels of motion. This may be used to improve scan efficiency saving time and money and increasing throughput and minimizing discomfort to the patient.

In this study, three of the FIDnav motion scores were computed relative to the previous repetition time. While this provided a sensitive metric to detect most pediatric head motion patterns in this study, one limitation is that a single abrupt motion resulting in repositioning of the head, would yield a relatively lower motion score, while adversely affecting diagnostic image quality. This may explain why some of the non‐diagnostic images were incorrectly identified with these integrated FIDnav motion metrics. Computing the percentage change relative to the baseline FIDnav signal performed less well due to drift in the FIDnav time course, which would require correction with a linear regression model.^26^ Another potential limitation is that FIDnavs are not uniquely sensitive to rigid‐body head motion, as they can detect signal changes related to swallowing or coughing, as well as changes in the local magnetic field that can be induced by arm motion (eg, subject scratching their nose or adjusting video goggles) and deep breathing.^36^ Together, these may account for some false positive scans in this study.

### 4.4 Practical applications

FIDnav‐based motion scores may be used to provide feedback to the operator to identify non‐diagnostic scans before the acquisition is complete or to trigger a prospective correction strategy to steer the FOV after detection of a motion event.^30^ FIDnav motion information may also be used retrospectively to identify motion‐corrupted data to improve the reconstruction.^37^ Real‐time feedback of motion information could also be given directly to the subject, for example, using a visual display that turns from green to orange to red as the motion score approaches the threshold and/or that interferes with movie‐watching while motion is detected. Such real‐time visual feedback has proven to be effective in increasing subject compliance, particularly in younger children.^11^, ^38^

As well as detecting non‐diagnostic scans in clinical studies, FIDnav motion monitoring may also be applied in the research setting, where the use of sedation is usually not an option. Research MRI scans are often longer in duration and acquire higher resolution image data, which is more sensitive to the effects of small motions.^39^ Head motion has also been shown to lead to systematic biases in derived morphological and functional measures.^40^, ^41^ For instance, significant (0.7%/mm/min) decreases in cortical gray matter volume and thickness estimates have been observed with increasing head motion, even in images with only mild motion degradation.^42^ Low‐impact FIDnav‐based motion metrics could help control and quantify the confounding effects of head motion when interpreting variations across groups or between individuals to avoid mistaking systematic bias for pathologic effects eg brain atrophy. For example, an integrated FIDnav motion score could be included as a nuisance regressor or used to match levels of motion between groups to increase the validity of cross‐sectional and longitudinal comparison studies.^41^, ^43^

### 4.5 Extension to other sequences and contrasts

In this work, we investigated FIDnav motion monitoring in a T_1_‐weighted MPRAGE sequence; however, the proposed approach is easily extendable to other sequences and contrast mechanisms. FIDnav measurements are dependent on various factors, including the acquisition parameters (eg, flip angle), coil configuration and field strength, which may require adjustment of the optimal threshold for motion detection. Several variants of the protocol were used in this study including water‐excitation and post‐contrast imaging. While water excitation should render the sequence less sensitive to motion (due to the absence of high‐intensity fat signal), the use of different excitation pulse types or contrast did not appear to introduce large discrepancies in the measured FIDnav signal and exclusion of these subjects did not substantially alter the correlation between FIDnav motion scores and radiologic evaluation of image quality (Supporting Information Table S1).

For 2D scans, FIDnavs may be inserted after the RF excitation pulse and slice rewinder, yielding a FIDnav measurement per coil and slice for each TR, which may be combined into a single metric by computing the mean or median over maximally changing slices.^29^ Motion detection in 2D may be even more sensitive due to the effects of out‐of‐plane motion on the measured signal. Alternatively, a stand‐alone FIDnav module with its own slab‐selective RF excitation pulse may be applied outside of each excitation/readout block of the host sequence to avoid interfering with timing and contrast characteristics.^27^ This also allows the FIDnav to be oriented independently of the host sequence, for example, the slab could be positioned to maximize sensitivity to rigid‐body head motion while minimizing detection of swallowing and deep breathing.

### 4.6 Study limitations

In this study, the sensitivity and specificity of four FIDnav motion detection algorithms were evaluated against radiologic evaluation of image quality. The FIDnav coil combination algorithms tested were not exhaustive, but were chosen based on previously proposed strategies in the motion detection literature.^26^, ^27^, ^28^ This study only considers one type of scan (T_1_‐weighted MPRAGE), acquired at 3T using a 32‐channel head coil; however, this is representative of a clinical scan acquired in many brain MRI protocols. The FID‐navigated MPRAGE was typically the last sequence in the protocol, with protocol lengths ranging from approximately 30 minutes to 1 hour. The success rate of scans tends to drop with longer protocols in younger subjects, who have limited compliance.^35^ However, as most children aged seven and under are sedated for MRI, a larger proportion of subjects in this study were between 8 and 18 years old. As scans took place at an outpatient facility, most of these subjects were reasonably co‐operative, which skewed the distribution of image grades to higher values (Supporting Information Figure S2). Larger time savings would potentially be realized if a larger proportion of study subjects were incompliant.

### 4.7 Conclusions

Computing the integrated cross‐correlation between multi‐channel FIDnav signals has high sensitivity and specificity for detecting clinically relevant pediatric head motion in structural brain images. Motion detection with FIDnavs has potential to increase scan efficiency and patient throughput, and reduce costs, both by minimizing time acquiring non‐diagnostic information and by reducing the need for sedation.

## CONFLICT OF INTEREST

Tobias Kober is an employee of Siemens Healthineers.

## Supporting information


**FIGURE S1** Weighting function derived from compliant adult subjects used to compute the partition‐weighted FIDnav motion score. The mean weighting function derived from pediatric subjects with high‐quality images (ranked as Grades 4 & 5) is shown for comparison
**FIGURE S2** Distribution of expert‐rated image grades across different age groups: (A) 0‐7 years (N = 12); (B) 8‐15 years (N = 51); (C) 16‐18 years (N = 39). The larger distribution of images ranked as Grades 4 or 5 with increasing age shows that older subjects are less motion‐prone
**TABLE S1** Spearman correlation coefficients between integrated FIDnav motion scores and expert‐rated image quality, computed across all subjects, and excluding post‐contrast examinations and scans with water excitation
