# Supplementary material for: Free induction decay navigator motion metrics for prediction of diagnostic image quality in pediatric MRI
Source: Magn Reson Med. 2021 Jan 6;85(6):3169–81. doi: 10.1002/mrm.28649 (PMC7904595; doi:10.1002/mrm.28649)
Supplement: Supplementary file 1 — FIGURE S1 Weighting function derived from compliant adult subjects used to compute the partition‐weighted FIDnav motion score. The mean weighting function derived from pediatric subjects with high‐quality images (ranked as Grades 4 & 5) is shown for comparison FIGURE S2 Distribution of expert‐rated image grades across different age groups: (A) 0‐7 years (N = 12); (B) 8‐15 years (N = 51); (C) 16‐18 years (N = 39). The larger distribution of images ranked as Grades 4 or 5 with increasing age shows that older subjects are less motion‐prone TABLE S1 Spearman correlation coefficients between integrated FIDnav motion scores and expert‐rated image quality, computed across all subjects, and excluding post‐contrast examinations and scans with water excitation [file MRM-85-3169-s001.docx]

**Supporting Information**


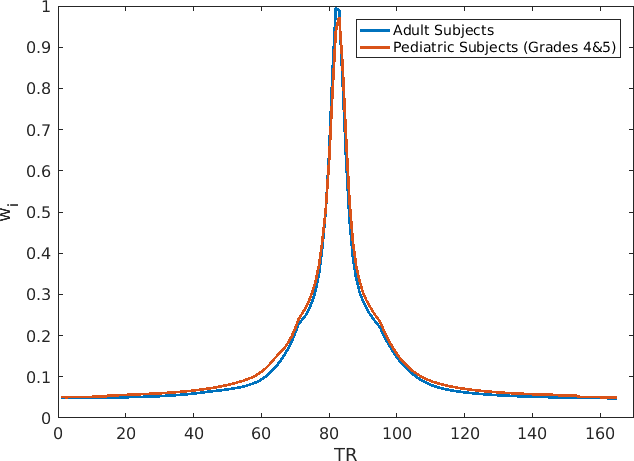


**Figure S1.** Weighting function derived from compliant adult subjects used to compute the partition-weighted FIDnav motion score. The mean weighting function derived from pediatric subjects with high-quality images (ranked as Grades 4 & 5) is shown for comparison.

**Table S1.** Spearman correlation coefficients between integrated FIDnav motion scores and expert-rated image quality, computed across all subjects, and excluding post-contrast examinations and scans with water excitation.

|  | $FIDnav$ (s^-1^) | | | | $wFIDnav$ (s^-1^) | | | |
| --- | --- | --- | --- | --- | --- | --- | --- | --- |
|  | $FIDnav_{\Delta ref}$ | $FIDnav_{\Delta}$ | $FIDnav_{\Delta max}$ | $FIDnav_{CCCC}$ | $FIDnav_{\Delta ref}$ | $FIDnav_{\Delta}$ | $FIDnav_{\Delta max}$ | $FIDnav_{CCC}$ |
| All subjects (*N* = 102) | 0.22 | 0.60 | 0.56 | 0.57 | 0.25 | 0.59 | 0.54 | 0.55 |
| Excl. Post-Contrast  (*N* = 90) | 0.16 | 0.61 | 0.58 | 0.55 | 0.21 | 0.60 | 0.56 | 0.53 |
| Excl. Water Excitation  (*N* = 76) | 0.03 | 0.71 | 0.67 | 0.61 | 0.01 | 0.69 | 0.64 | 0.58 |


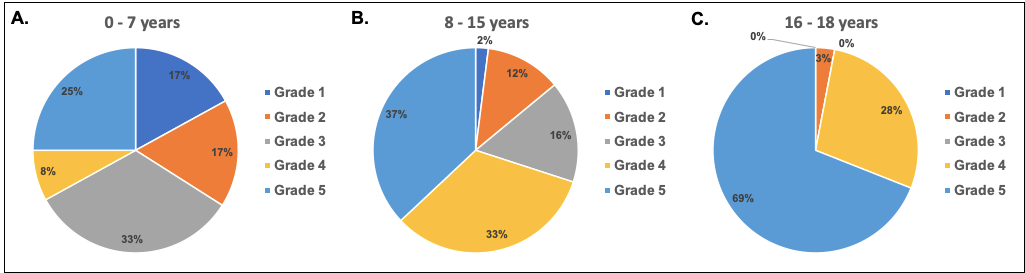


**Figure S2.** Distribution of expert-rated image grades across different age groups: (A) 0–7 years (*N* = 12); (B) 8–15 years (*N* = 51); (C) 16–18 years (*N* = 39). The larger distribution of images ranked as Grades 4 or 5 with increasing age shows that older subjects are less motion-prone.
